# Recent Research on Gastrointestinal Carcinoma

**DOI:** 10.3390/cancers13020333

**Published:** 2021-01-18

**Authors:** Giulia Rovesti, Giorgia Marisi, Andrea Casadei-Gardini

**Affiliations:** 1 Department of Oncology and Hematology, Division of Oncology, University of Modena and Reggio Emilia, 41125 Modena, Italy; giulia.rovesti@gmail.com; 2 Biosciences Laboratory, Istituto Scientifco Romagnolo per lo Studio e la Cura dei Tumori (IRST), IRCCS, 47014 Meldola, Italy; giorgia.marisi@irst.emr.it; 3 Department of Medical Oncology, Università Vita-Salute, San Raffaele Hospital IRCCS, 20019 Milan, Italy

This series of 10 articles (eight original articles and two reviews) is presented by international leaders in gastrointestinal cancer research. As highlighted by this Special Issue, research is currently moving in different directions (e.g., genetics, epigenetics, and metabolomics), leading to remarkable advances in diagnosis, treatment, and prognosis estimation, with particular attention being paid to the personalization of approaches. 

Regarding locally advanced gastric cancer treatment, Monti et al. present the results of the GASTRODOC study, an Italian, multicenter, open-label, phase-II trial aimed at evaluating whether neoadjuvant treatment is superior to perioperative treatment in terms of the percentage of completed cycles of chemotherapy (docetaxel, oxaliplatin, and capecitabine) [1]. Although not as prevalent as the conventional subtype, early-onset gastric cancer represents a proper model for studying the genetic background of this tumor. Through next-generation sequencing technology and, therefore, in a high-throughput way, Machlowska and colleagues assess mutation profiling for the most frequent alterations in gastric cancer development, revealing that early-onset gastric cancer has a different mutation frequency profile in certain genes if compared to the conventional subtype [2]. Moving from genomics to epigenomics, testing of methylated RUNX3 through liquid biopsy with a novel and analytically sensitive technique (combined restriction digital PCR (CORD) assay) appears to be an alternative and effective screening strategy to detect gastric cancer even at an early stage [3]. Long noncoding RNAs (lncRNAs) represent other important regulators of the epigenetic status of the human genome, and their dysfunction plays a critical role in carcinogenesis of several cancers, such as colorectal cancer. The role of a novel lncRNA, named LOC441461, is extensively elucidated by Wang et al., who not only highlight its oncogenic role and its significantly higher expression in colorectal cancer (CRC) but also provide insights into its application for treatment purpose [4]. Interestingly, preoperative short-course radiotherapy, among the standard treatments for rectal cancer, may induce morphological features simulating dysplasia in the adjacent normal mucosa, possibly resulting in overtreatment of a patient if misinterpreted. Zanelli and coauthors provide a comprehensive morphological description of radiation-induced abnormalities; through next-generation sequencing (NGS) analysis, they support the findings that radiation-induced “dysplastic-like” features are not genetically transformed [5]. It is well known that, in the era of precision medicine, molecular-biology-driven treatment has a pivotal role in improving patient selection and outcomes, thus avoiding unnecessary toxicities and providing better allocation of economic resources. In this context, Lai and collaborators provide a comprehensive review of the molecular biomarkers currently driving tailored treatment for metastatic CRC patients and shed light on those that might become relevant in the near future [6]. Although further studies are needed, variations in FGF2 levels might represent a potential biomarker in patients who are more likely to gain benefit from bevacizumab and other anti-VEGF (vascular endothelial growth factor) drugs. Notably, FGF2 levels seem to reach their highest value just before disease progression during bevacizumab treatment, possibly leading to studies focused on FGF2 inhibition in second-line therapy or subsequent lines of treatment [7]. Shifting the concept of personalized therapy and the need for predictive biomarkers to the setting of advanced neuroendocrine tumors (NETs), activation of the mTOR pathway and, in particular, the expressions of phosphorylated (p)-mTOR and p70S6-kinase (S6K), a downstream effector of mTOR, can predict better outcomes in patients with NETs of various origins treated with everolimus [8]. An altered tumor metabolism is among the hallmarks of cancer, and thus, the large-scale study of metabolites, so-called metabolomics, is gaining more and more attention. Proton nuclear magnetic resonance (1H-NMR) serum metabolomics profiling coupled with multivariate data analysis can define a metabolic signature able to discriminate early hepatocellular carcinoma (HCC) from advanced HCC patients with a cross-validated accuracy that is approximately 100% [9]. The review by Indellicato et al. is paradigmatic of the importance of research and of its role in pushing forward even the most established knowledge, since it might promise fruitful advances. Indeed, 30 years after the first use of CA19.9 and its related antigens as tumor markers, circulating Lewis a and Lewis b have been shown to be promising candidates for monitoring CA19.9-negative pancreatic cancer patients [10]. 

In conclusion, this Special Issue provides updated knowledge in the field of gastrointestinal cancer research, addressing the topic from diagnosis to treatment and at different levels, reflecting the unique and multifaceted landscape of modern cancer research.
